# Design choices made by target users for a pay-for-performance program in primary care: an action research approach

**DOI:** 10.1186/1471-2296-13-25

**Published:** 2012-03-27

**Authors:** Kirsten Kirschner, Jozé Braspenning, JE Annelies Jacobs, Richard Grol

**Affiliations:** 1 Scientific Institute for Quality of Healthcare, Radboud University Nijmegen Medical Centre, P.O. Box 9101, Nijmegen, HB 6500, The Netherlands

## Abstract

**Background:**

International interest in pay-for-performance (P4P) initiatives to improve quality of health care is growing. Current programs vary in the methods of performance measurement, appraisal and reimbursement. One may assume that involvement of health care professionals in the goal setting and methods of quality measurement and subsequent payment schemes may enhance their commitment to and motivation for P4P programs and therefore the impact of these programs. We developed a P4P program in which the target users were involved in decisions about the P4P methods.

**Methods:**

For the development of the P4P program a framework was used which distinguished three main components: performance measurement, appraisal and reimbursement. Based on this framework design choices were discussed in two panels of target users using an adapted Delphi procedure. The target users were 65 general practices and two health insurance companies in the South of the Netherlands.

**Results:**

Performance measurement was linked to the Dutch accreditation program based on three domains (clinical care, practice management and patient experience). The general practice was chosen as unit of assessment. Relative standards were set at the 25^th^ percentile of group performance. The incentive for clinical care was set twice as high as the one for practice management and patient experience. Quality scores were to be calculated separately for all three domains, and for both the quality level and the improvement of performance. The incentive for quality level was set thrice as high as the one for the improvement of performance. For reimbursement, quality scores were divided into seven levels. A practice with a quality score in the lowest group was not supposed to receive a bonus. The additional payment grew proportionally for each extra group. The bonus aimed at was on average 5% to 10% of the practice income.

**Conclusions:**

Designing a P4P program for primary care with involvement of the target users gave us an insight into their motives, which can help others who need to discuss similar programs. The resulting program is in line with target users' views and assessments of relevance and applicability. This may enhance their commitment to the program as was indicated by the growing number of voluntary participants after a successfully performed field test during the procedure. The elements of our framework can be very helpful for others who are developing or evaluating a P4P program.

## Background

International interest in pay-for-performance (P4P) initiatives to improve quality of health care is growing. Despite the proliferation of P4P programs, the evidence to support their use is still inconclusive [1,2]. One of the reasons may be the differences between P4P programs. Incentives in current programs vary in terms of number and type of indicators, professional standards and quality domains (clinical care, patient experience, organisation of care) [3-7]. The size of the incentive and the unit of assessment in P4P programs can influence their effectiveness [8]. Experiences with different P4P programs led to a framework for design choices regarding the P4P approach. Three essential framework components to design a P4P program can be distinguished: performance measurement, appraisal and reimbursement [9-12]. The performance measurement should include valid and reliable indicators that make sense to the target group. Appraisal in a P4P program means defining the unit of assessment and the performance standards, but also describing the analysis and interpretation of the data. Based on the analysis and interpretation of the data a reimbursement system can be built [10].

Another remarkable feature of current P4P programs is that they are mostly designed and implemented top-down by policy makers and managers [13]. P4P programs can be seen as an innovation in care, and it is known that the sustainability of an innovation can be improved by involving target users [14]. It has also been suggested to involve target users in the developmental process of a P4P program, because this can contribute to the effect of incentivized indicators [15,16]. A more bottom-up procedure in designing a P4P program may improve its future implementation and its effectiveness.

Evaluation of the involvement of target users in de decisions about the P4P program may contribute to the growing field of P4P research. One may assume that involvement of health care professionals in the goal setting and methods of quality measurement and subsequent payment schemes may enhance their commitment to and motivation for P4P programs and therefore the impact of these programs. Nevertheless, you have to reckon with conflicts of interest when involving target users. Therefore it is important to develop the P4P program in a systematic way, such as the Delphi procedure [17]. The perspectives of all target users become distinct, and the decisions made are transparent for the target users. The aim of our study was to design a P4P program using a bottom-up procedure, in which the different options for performance measurement, appraisal and reimbursement were discussed by the target users in a systematic consensus procedure. We will present this bottom-up process of development of the P4P program and its resulting design.

## Methods

### The design options in the P4P framework

We searched the literature for relevant elements for our P4P program, to be discussed by the target users. Table 1 gives an overview of the elements and design options.

**Table 1:** Elements of the P4P program, design options and choices

Component	Elements	Design options	Design choices P4P program
**Performance measurement**	**Performance indicators**		
	
	*Domains, subjects and indicators*	Selection of:- Clinical care (diabetes, asthma, COPD, cardiovascular risk management, influenza vaccination, cervical cancer screening, prescribing acid suppressive drugs and antibiotics)- Practice management (infrastructure, team, information, quality and safety)- Patient experience (experience with general practitioner and organisation of care)	Selected indicators for:- Clinical care: diabetes (n = 9), asthma (n = 4), COPD (n = 5), cardiovascular risk management (n = 9), influenza vaccination (n = 2), cervical cancer screening (n = 1), prescribing antibiotics (n = 2)- Practice management: infrastructure (n = 7), team (n = 8), information (n = 3), quality and safety (n = 4)- Patient experience: experience with general practitioner (n = 16) and organisation of care (n = 11)
	
	*Period of data collection*	Data collection for all three domains each year vs. a trimmed-down version of the program	At baseline measurement of clinical care, practice management, patient experience; In following years only clinical care and patient experience

**Appraisal**	**Unit of assessment**	Individual GP vs. general practice vs. larger organisational unit	General practice
	
	**Performance standards**	• Absolute vs. relative standards • Same standards vs. different standards for indicators/subjects	• A relative standard set at the 25^th^ percentile of group performance • Different standards for indicators
	
	**Analysis and interpretation of performance data**		
	
	*Weighing the domains*	Different weights vs. same weight	Clinical care : practice management : patient experience 2:1:1
	*Weighing the indicators*	Different weights vs. same weight	Same weight for all indicators
	
	*Calculations*	• Separate scores for each domain vs. one overall domain-score • Calculations for quality level and/or improvement of performance	• Separate scores for each domain • Calculations for both quality level and improvement of performance
	
	*Weighing the quality scores*	Different weights vs. same weight for quality level and improvement of performance	Quality level : improvement of performance 3:1
	
	*Differentiation of quality scores*	4 levels vs. 5-7 levels vs. 8-10 levels	7 levels
	
	*Feedback*	• Benchmark: median vs. best practice (75^th^ or 90^th^ percentile) vs. a combination • Risk adjustment: indirect vs. direct correction • One-step procedure vs. two-step procedure	• 25^th^ percentile, median, 75^th^ percentile • No risk adjustment • Two-step procedure

**Reimbursement**	**Financial rewards**		
	
	*Payment*	Money vs. human resources vs. sabbatical leave vs. a combination	Money
	
	*Size of the bonus*	5000 Euros to 10000 Euros (5-10% practice income) on average per practice (depending on practice size) → appropriate or not?	5000 Euros to 10000 Euros on average per practice (depending on practice size)Baseline: A maximum of euro 6.89 on average per patient*Following years: A maximum of euro 2.88 on average per patient*
	
	*Spending the bonus*	No obligations vs. obligations (spending for practice with or without pre-set goal) vs. a combination	No obligations

* A patient whose health insurance company was a sponsor of the P4P program

The *performance indicators *covered three domains, clinical care, practice management and patient experience, and were derived from the Dutch National Accreditation Program for general practices [18]. The target users were asked whether these three domains, the subjects and the indicators were appropriate for the P4P program. For clinical care the target users could choose from indicators for diabetes, COPD, asthma, cardiovascular risk management, influenza vaccination, cervical cancer screening and prescribing acid suppressive drugs and antibiotics. For practice management, which is measured with the validated Visitation Instrument Practice management (VIP) [19], they could approve various indicators for infrastructure, team, information, and quality and safety. The indicators for patient experience to agree on were based on the internationally validated EUROPEP instrument [20], which evaluates both the general practitioner and the organisation of care. Furthermore, target users could decide on collecting data for all three domains each year versus a trimmed-down version of the program.

The *appraisal *and *reimbursement *elements and options to be discussed were derived from the literature [1,8,10,11]. The following design elements of P4P programs were described: unit of assessment, performance standards, analysis and interpretation of performance data, and financial rewards. The options for the unit of assessment, either the general practitioner, the general practice or a larger organisational unit, were presented together with evidence that the smaller the unit the stronger the stimulation of quality improvement [1], and the practical consideration that the general practice is the unit of assessment within the Dutch National Accreditation program. The options presented for performance standards were either absolute or relative performance standards [10]. Most existing programs are based on absolute standards [1,8,21]. The target users were asked whether performance standards should vary between indicators/subjects. Some indicators might need lower minimum standards because they are more difficult to reach than others. Concerning the analysis and interpretation of performance data the options were to weight domains and indicators either differently or to weigh them equally. In the Quality and Outcomes Framework (QOF), for instance, performance on clinical indicators receives more weight than practice management or patient experience [7]. For calculating quality scores options were to either calculate a quality score for each domain separately or to calculate one overall domain-score. Moreover the target users could choose whether both the quality level and the improvement of performance should be incentivized and whether to weigh the scores differently or equally. A combination of incentives for both the quality level and improvement of performance will encourage both low and high performing providers to improve quality [1,16]. In order to link a bonus to the quality, quality scores need to be differentiated into levels. The options given were: 4 levels (quartiles), 5 to 7 levels, or 8 to 10 levels. The more levels, the more smaller improvements will be worth the investment. For the feedback a discussion was started on a proper benchmark and on risk adjustment. The options presented for a benchmark were the median, the best practice (75^th^ percentile or 90^th^ percentile) or a combination. Improvement can best be stimulated by feedback in a reachable range [22], thus practices with relatively low scores are stimulated by the average of the peer group, and practices with high scores by best practices. Comparing practices with others without appropriate risk adjustment can be misleading. Risk factors include patient demographic and/or clinical factors, which can influence outcomes of care. The target users had to decide on risk adjustment of the indicator scores, which is either to adjust the benchmark (indirect correction) or the indicator scores (direct correction). Concerning the feedback the target users could choose either a one-step procedure or a two-step procedure. In the one-step procedure practices receive feedback and bonus together. In the two-step procedure practices first receive feedback, and receive the bonus only after they have had the opportunity to respond to their feedback.

Concerning the *reimbursement *the options for the method of payment were either money, human resources, a sabbatical leave or a combination of these. For the size of the bonus we asked the target users whether an average bonus of 5000 to 10000 Euros (depending on practice size), which is on average 5-10% of the practice income, would be appropriate. In other P4P programs the size of the bonus varied between US$2 per patient and US$ 10000 per practice [23]. The size of the bonus should not be too small as this may limit the effects, but neither should it be too high because of unintended consequences like gaming [21,24,25]. The options for spending the bonus were either without obligations or with obligations (spending for the practice related to a goal, possibly preset) or a combination of these options.

### Study design

**Figure 1:**
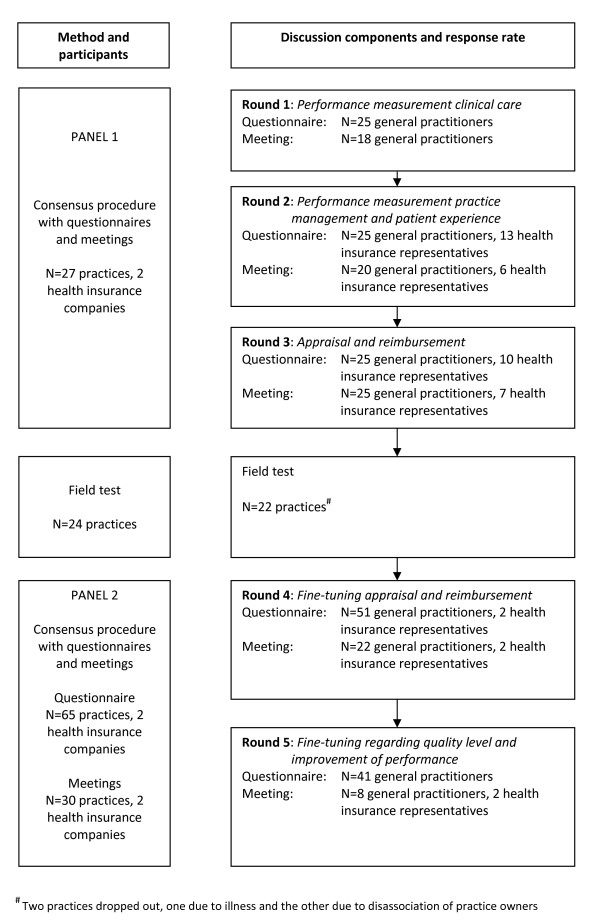
**Procedure design selection of a P4P program by target users**.

An action research approach [26] was applied with participation of future target users in the development of the P4P program. To reach consensus an adapted Delphi procedure [27] was used in two panels of target users. (Figure 1) The target users in question were general practitioners (GPs) and payers (representatives of health insurance companies). General practices in the South of the Netherlands were invited by the two regional health insurance companies to participate voluntarily in this P4P experiment. We aimed at participation of 20 to 25 general practices, and at least one representative of each health insurance company. To achieve consensus on the P4P design, two rounds were organized to discuss the methods of performance measurements (one on clinical care, and one on practice management and patient experience) and one round to discuss the methods of appraisal and reimbursement. The participating practices were also invited to volunteer in a field test in which data were collected based on the previous choices for the measurement of clinical performance, practice management and patient experiences. Feedback to the practices was delivered and the resulting bonus was paid according to the system agreed on. After the field test the panel was extended with general practices that were also willing to participate in this P4P experiment. In this second panel we discussed the methods of appraisal and reimbursement based on the results of the field test (round four) and the design options regarding quality level and improvement of performance (round five) to fine-tune the P4P program.

### Consensus procedure

In each round a written questionnaire with the design options for the P4P program was sent to the target users two weeks before the planned meeting. In the questionnaire they were provided with background evidence on the options as described in the section 'The design options in the P4P framework' and they were asked to make a choice. Each meeting started with explaining the aim of the discussion and feedback on the results of the questionnaires. All design options were discussed, but for the performance indicators the project team decided not to discuss indicators with high consensus, defined as less than 30% or more than 70% in favour. At the end of each meeting the panel members completed the same questionnaire again. The decision rule for inclusion of clinical indicators was set at more than 70% in favour, and for the other design options a majority rule was applied.

All panel meetings were held in the region in question to enhance participation. Payers and GPs attended the same discussion meetings which lasted 2 hours. The general practices in the first panel received 1500 Euros for participating in the panel as well as in the field test. Each GP in the second panel received 100 Euros for attending the meetings.

## Results

### Study population

The number of general practitioners and health insurance representatives that filled in the questionnaires and attended the meetings for the specific panels are presented in Figure 1. The number of GPs that could attend the meetings in round four and five were restricted to 30 due to the large number of practices that voluntarily participated in the P4P program. In panel 1 the response rate for the GPs was on average 93% for the questionnaires and 78% for the meetings, and in panel 2 71% and 50% respectively. The health insurance representatives decided to leave the discussion on the performance indicators to the experts (GPs). They participated amply in panel 1 and their participation decreased in panel 2.

### Design choices

The successive panel procedures and the field test resulted in a P4P program which is presented in Table 1.

### Performance measurement

The target users thought clinical care, practice management and patient experience to be appropriate domains for the P4P program, as well as the subjects within these domains (see Table 1). Some GPs remarked that the *clinical conditions *to be assessed were mainly focused on chronic care, though GP care comprises much more. Especially communication skills were missed. Although patient experiences were to be assessed, some GPs stated that communication was not reflected enough in the indicators. The GPs also discussed the fact that choosing indicators would result in a certain focus that could distract them from the more general goal of quality improvement. Practices may concentrate their performance on the indicators from the P4P program. It was proposed that in the long-term a large set of indicators will be needed. Then the P4P program could have different sets for different years. Some GPs even suggested that the practice should not be aware of the existing set. For clinical care, GPs were convinced that the outcomes were a mixed result of patient and doctor's performance. It was therefore decided that the payment should be based on the process measures only, but the GPs would like to receive feedback on the outcome indicators as well. So, data for both process and outcome indicators were collected. Although the health insurance representatives stated they would leave the decisions on clinical care options to the GPs, they joined the discussion in the meetings on the prescription indicators. The prescription indicators were highly valued by the health insurance representatives. The GPs questioned these indicators which resulted in not including the acid suppressive drugs indicators in the program, and including indicators on prescribing antibiotics. Some items of *practice management *were excluded due to their estimated low correlation with the quality of practice management such as financial accounting. The EUROPEP instrument, consisting of 23 items measuring *patient experience*, was supplemented with four items regarding the possibility to ask for a longer consultation, accessibility by phone, getting an appointment with your own doctor and an accessible procedure for complaints. All selected quality indicators can be found in additional file 1.

The target users agreed that at *baseline*, data should be collected for all three domains. For the *years to follow *the data collection for the practice management domain was judged to be unnecessary as it is not likely that this will change substantially over two or three years and as the data collection in this domain is very labour-intensive.

### Appraisal

The general practice was chosen as *unit of assessment*, because in the context of the P4P program the incentive would be targeted at this level. Since the data on clinical performance were collected at individual GP level, practices asked to receive feedback at the level of the GP as well. *Relative standards *for determining the level of the incentive were preferred over absolute standards by most target users. Setting an absolute standard for performance was considered too arbitrary. They preferred performance standards for both indicators and subjects, but as this would not contribute to the clarity of the calculations it was decided to restrict it to the indicators only. They agreed to set the relative performance standard at the 25^th^ percentile of each indicator. This responded to the preference of the panel members to vary the performance standards between the indicators. The target users thought that all individual indicators should receive the same *weight *because a good criterion for differentiating was lacking. However, they decided that clinical care should receive double the weight of practice management and patient experience (2:1:1) because clinical care is the major domain of quality of care. The data from the 22 practices in the field test showed that the quality scores should be calculated separately for each domain because otherwise the performance on clinical care would dominate the overall quality score. Having data available for two or more years made it possible to calculate improvement of performance as well. Panel 2 decided to reward *quality level as well as improvement *of performance in a ratio of 3:1 for the bonus payment in the next year. In that case practices with high scores would receive a bonus for delivering quality and practices with low scores would be stimulated to improve. In the following years two separate scores will be calculated for each practice; one on quality level and one on improvement of performance for the three domains clinical care, practice management and patient experience. The panel thought of the P4P program as a three years cycle in which practice management was only measured at the beginning of each cycle. The users preferred to have a detailed division in levels of quality scores to make small differences in quality visible and to make it easier to achieve next levels. The range of quality scores of all participating practices were therefore divided into *seven equal levels (*relative 'thresholds'). Practices that do not improve will be rated in level 0 (no bonus) and practices showing improvement will be rated in one of six levels with a differentiation in bonus accordingly. For feedback the target users preferred a *benchmark *with the 25^th^ (minimum standard), 50^th^ and the 75^th^ percentile because that would give them a good overview and stimulate practices at the bottom as well as at the top. *Risk adjustment *for the process indicators was discussed. The target users preferred stratification, which is a comparison with a benchmark consisting of comparable practices instead of correction of their own data. However, stratification would require a large sample of practices, so we decided not to include this aspect in the experiment. Following the experiences with the field test, a *two-step procedure *was chosen by the GPs. Practices will receive feedback (indicator scores and benchmark) and the bonus after they have had the opportunity to respond to their feedback. The feedback was accompanied with clear information on the calculation procedure.

### Reimbursement

The discussion on the type of financial reward resulted in the conclusion: "Money is the best method, because money can buy you anything". The target users agreed on a bonus that was 5 to 10% of practice income with a minimum of 0 Euros and a maximum of 15000 Euros. None of the users indicated this amount was too high and half of them thought this was too low. However, agreement was reached with the argument that the proposed size of the bonus was in line with bonuses paid in trade and industry. A decline in the bonus for the years to follow had to do with the budget of the health insurance companies who rationalized it by arguing that the data collection was most labour intensive at the start of the project. According to the discussion on the appraisal we need two formulas to calculate the bonus, one on the quality level and one on quality improvement level. The quality improvement level can only be calculated after the first year. The formulas are:

$$\begin{array}{l} \text{Bonus practice}\left(\text{i}\right)\text{on quality level}\left(\text{QL}\right)=\text{3}(\text{2}*\text{clinical care QL}\left(\text{x}\right)\;+\;\text{patient experience QL}\\ \left(\text{y}\right)\;+\;\text{practice management QL}\left(\text{z}\right))*\text{number of patients in practice insured by payers} \end{array}$$

$$\begin{array}{l} \text{Bonus practice}\left(\text{i}\right)\text{ on quality improvement level }\left(\text{QIL}\right)\;=\;(\text{2}*\text{clinical care QIL}\left(\text{x}\right)\;+\;\text{patient experience QIL}\\ \left(\text{y}\right))\;*\text{ number of patients in practice insured by payers} \end{array}$$

The exact bonus at baseline and in following years is presented in Table 2. The maximum bonus in year 1 is 6.890 Euros per 1000 patients (which is on average 7500 Euros for a practice with 2350 patients) and in the following years 2.880 Euros. Target users found that formulating explicit criteria for spending the bonus was not necessary. Rewarding good quality versus penalizing poor quality was discussed in the panel as well, but proved to be not applicable at this stage of the P4P program.

**Table 2:** Bonus per patient for the first year and the following years for each domain and quality (improvement) level

Baseline bonus for clinical care, practice management and patient experience per patient								
	Quality score	0	1	2	3	4	5	6

Clinical care	Quality level	€ 0	€ 0.83	€ 1.33	€ 1.87	€ 2.37	€ 2.95	€ 3.45

Practice management	Quality level	€ 0	€ 0.41	€ 0.66	€ 0.94	€ 1.19	€ 1.47	€ 1.72

Patient experience	Quality level	€ 0	€ 0.41	€ 0.66	€ 0.94	€ 1.19	€ 1.47	€ 1.72

**Bonus in following years for clinical care and patient experience per patient**								

	Quality score	0	1	2	3	4	5	6

Clinical care	Quality level	€ 0	€ 0.25	€ 0.50	€ 0.75	€ 1.00	€ 1.25	€ 1.50
	
	Quality improvement	€ 0	€ 0.07	€ 0.14	€ 0.21	€ 0.28	€ 0.35	€ 0.42

Patient experience	Quality level	€ 0	€ 0.12	€ 0.25	€ 0.37	€ 0.50	€ 0.62	€ 0.75
	
	Quality improvement	€ 0	€ 0.03	€ 0.07	€ 0.10	€ 0.14	€ 0.17	€ 0.21

## Discussion

P4P proves to be a complex innovation and knowledge needs to be acquired over time [10]. Assuming a greater probability of acceptance of P4P programs and subsequent quality improvement, our study contributes to this field by describing the design choices of target users when they themselves are involved in developing a P4P program. We succeeded in involving the target users in the lively discussions about design options. They were very much involved in the discussions and in the field test; the response rate in the panels was high. We managed to reach consensus and to define a P4P program for primary care in the Netherlands.

In line with other P4P programs our target users selected performance indicators for clinical care, practice management and patient experience. It was not surprising that the chronic diseases were chosen for the program concerning the attention for these diseases and concerning the health care costs due to these diseases. However, our program seems to be more balanced compared to other programs with regard to the position of patient experiences in the program [3,5-7]. GPs indicated that they wanted the patient to be more central in the program because patient communication is a core task. Nevertheless, they wanted clinical care to be weighted more heavily than patient experience to reduce the chance of being solely judged on patient experiences. Here the consequences of the choices seem to overrule the principles.

Mostly, P4P programs are designed and implemented top-down by policy makers and service managers [13]. In our study both GPs and health insurance companies were involved in the development of the program. Interestingly, the health insurance representatives did not want to discuss the content of clinical care and allowed the GPs to decide on this domain. In other programs the payers had a more decisive role in the development of a P4P program or were not involved in any way. Though the effectiveness of P4P programs is still inconclusive, we assume that our approach enhances the commitment and motivation of general practices and therefore the impact of our program.

The target users had a realistic estimate of the required size of the bonus in order to achieve a quality stimulus. According to the target users a bonus of on average 5-10% of practice income was considered to be appropriate. The target users were aware of the risk of gaming when the incentive is too high [21,24,25]. Our bonus is much smaller than the incentive in the UK which makes up approximately 25% of GPs' income [4].

The target users opted for relative P4P standards. Until now, programs mainly base their incentives on absolute performance standards [1,3,5,6,8,28]. An advantage of relative standards is that health insurers can remain within their budget. This is in contrast to the UK P4P program, for example [29]. Furthermore quality scores of all participating practices were divided into seven levels. These series of tiered thresholds have attainable goals for each practice; a known effective stimulus for changing behaviour [30]. According to the target users both quality level and improvement of performance need to be incentivized. This will stimulate practices with a high performance as well as practices with a low performance [1]. This is in contrast with other P4P programs in which nearly always good performance instead of improvement is rewarded [11].

### Strengths and limitations of the study

The strength of our study lies in its developmental process, assuming a greater probability of acceptance of the program and subsequent quality improvement. Involving the target users resulted in good discussions and consensus about the design options. The field test was performed successfully as part of the procedure. Many practices participated in the field test as well as in the panels, which resulted in a reasonably balanced P4P program.

This study has some limitations. First, due to time constraints patients were not included in the design of the P4P program. However, they had been previously involved in discussions about the objectives of the Dutch accreditation program, which was part of the initial framework of our program. Second, there is a drop in the number of participants in panel 2. A possible explanation is that the subjects we discussed in panel 2 were more restricted and detailed and therefore less attractive than those in panel 1. Third, practices could voluntarily register for this experiment, which may have resulted in overrepresentation of early adopters of a P4P program. It is important that the early majority as well as later on the late majority support the P4P program. To involve them in design choices that are acceptable and applicable is still a challenge.

### Strengths and limitations of the design choices

A strength of the design choices is the involvement of the target users. The behavioral change of the P4P program is therefore grounded in extrinsic (reimbursement) as well as intrinsic motivation. To stimulate the motivation further the feedback will be discussed within the practice supported by a facilitator.

The performance measures do not cover all aspects of general practice. Just stimulating the incentivized parts of the performance can result in a possible decline in quality of care of the non-incentivized aspect [31]. By discussing which aspects will be stimulated in the forthcoming period, we assume that this effect is somewhat lower in our P4P program.

GPs have decided that the outcome indicators on clinical care will not be incentivized, and the health insurance companies agreed. We have to study the effect of this decision on the outcome indicators. By incentivizing the process indicators an indirect effect is expected on the reported outcome measures, but that still has to be proven.

As in other P4P programs the focus of the clinical performance measures is on the chronic conditions. Policy makers show a lot of interest in the performance on these conditions, resulting in several improvement projects. This might have an effect on our baselines measures in which case the room for improvement decreases. In our effect study we will take into account the baseline measures to get more insight into this problem.

The relative thresholds might evoke calculating behavior, that is if no one improves the bonus will still be dived. The question is which practice will take this risk. This design choice introduces a prisoner's dilemma with unclear results. Although, based on the involvement of the participating GPs in quality of care we would assume that in this group the urge of improvement is larger. The sustainability of the relative thresholds can become tensed in a broader probably less involved group of GPs.

The health insurance companies decided together with the GPs on the available budget for the bonus. However, after the first bonus was allocated, the health insurance companies started the discussion on the bonus again. They suggested that the data collection was much easier in the following year, and therefore the bonus could be reduced. This lead to a lot of turbulence among the participating GPs. No consensus was reached, but the practices were still willing to participate. This is a demonstration of the inequity in the relation between payers and GPs that is hard to cover with a consensus procedure.

The sustainability of the P4P program is also stressed if the performance measures stay the same each cycle, because the indicators scores increase due to the P4P program to a certain optimum. This phenomenon has been described with the UK-QOF data [4]. To prevent this effect it was discussed that the P4P program will need constant trimming, recalibrating and balancing to ensure that the objectives are being met at the right costs and without too many unwanted effects. This means that subjects and/or indicators will be replaced with others when performance on the subjects reaches a certain level. This will also prevent a narrow focus on quality of care in general practice. The adjustments of the P4P program should again be based on discussions with the target users.

## Conclusions

By performing a procedure to involve target users in designing a P4P program for general practice, a detailed framework to define design choices was established. This framework as well as the insight into motives for design choices of the target users can be helpful for others who are developing or evaluating a P4P program. The resulting design resembled the P4P programs from other countries, but ours was also in line with target users' views and assessments of relevance and applicability. As already shown by the growing number of voluntary participants during the study, this may enhance general practitioner's commitment to the program.

## Competing interests

The authors declare that they have no competing interests.

## Authors' contributions

KK, the main investigator, carried out this study. She analyzed the questionnaires, organized the panel meetings and drafted this manuscript. JB, the project leader, was involved in all aspects of the study. AJ and RG participated in discussions about the reporting. All authors read and approved the final version of the manuscript.

## Pre-publication history

The pre-publication history for this paper can be accessed here:

http://www.biomedcentral.com/1471-2296/13/25/prepub

## Supplementary Material

Additional file 1 **The indicator set of the P4P program**. Click here for file
