# Supplementary material for: Design choices made by target users for a pay-for-performance program in primary care: an action research approach
Source: BMC Fam Pract. 2012 Mar 27;13:25. doi: 10.1186/1471-2296-13-25 (PMC3352266; doi:10.1186/1471-2296-13-25)
Supplement: Additional file 1 — The indicator set of the P4P program. [file 1471-2296-13-25-S1.DOC]

**The indicator set of P4P program**

**Clinical Care**

| **Diabetes** | |
| --- | --- |
| Information from the previous 12 months | |
| 1 | The percentage of patients with diabetes who have had three times a glucose measurement |
| 2 | The percentage of patients with diabetes who have a record of HbA1c |
| 3 | The percentage of patients with diabetes who have a record of the blood pressure |
| 4 | The percentage of patients with diabetes who have a record of total cholesterol |
| 5 | The percentage of patients with diabetes who use cholesterol medication |
| 6 | The percentage of patients with diabetes who have a record of serum creatinine testing |
| 7 | The percentage of patients with diabetes with a record of neuropathy testing |
| 8 | The percentage of patients with diabetes who have a record of retinal screening in the previous 24 months |
| 9 | The percentage of patients with a fully completed risk profile |

| **COPD** | |
| --- | --- |
| Information from the previous 12 months | |
| 1 | The percentage of patients with COPD in whom a spirometry has been done ever |
| 2 | The percentage of patients with COPD in whom a spirometry has been done in the previous 12 months |
| 3 | The percentage of patients with COPD with which there has been contact |
| 4 | The percentage of patients with COPD in whom there is a record of smoking status |
| 5 | The percentage of patients with COPD who smoke, whose notes contain a record that smoking cessation advice has been offered |

| **Asthma** | |
| --- | --- |
| Information from the previous 12 months | |
| 1 | The percentage of patients with asthma in whom a spirometry or a peak flow measurement has been done ever |
| 2 | The percentage of patients with asthma with which there has been contact |
| 3 | The percentage of patients with asthma in whom there is a record of smoking status |
| 4 | The percentage of patients with asthma who smoke, and whose notes contain a record that smoking cessation advice has been offered |

| **Cardiovascular risk management** | |
| --- | --- |
| Information from the previous 12 months | |
| 1 | The percentage of high risk patients whose notes have a record of blood pressure |
| 2 | The percentage of high risk patients whose notes have a record of total cholesterol or cholesterol ratio |
| 3 | The percentage of high risk patients with statins |
| 4 | The percentage of high risk patients whose notes record smoking status |
| 5 | The percentage of high risk patients who smoke, and whose notes contain a record that smoking cessation advice has been offered |
| 6 | The percentage of high risk patients with a fully completed risk profile |
| 7 | The percentage of patients with heart disease in anamnesis who are using anticoagulant drugs |
| 8 | The percentage of high risk patients whose notes record a glucose measurement |

| **Influenza vaccination** | |
| --- | --- |
| 1 | The percentage of vaccinated high risk patients in practice |
| 2 | The percentage of vaccinated patients of 65 years and older |

| **Cervical cancer screening** | |
| --- | --- |
| 1 | Women from target cohort screening whose notes record a cervical smear |

| **Antibiotics** | |
| --- | --- |
| Data from the previous 12 months | |
| 1 | Narrow-spectrum antibiotic cures in relation to all antibiotic cure prescriptions |
| 2 | Number of antibiotic prescriptions per 1000 patients |

**Practice management**

(answering scale yes/no)

| **Infrastructure** |  |
| --- | --- |
|  | Items |
| Presence of adequate space | 2 |
| Accessibility and availability | 3 |
| Presence of instruments for diagnosis and treatment | 11 |
| Presence of instruments for laboratory supplies | 8 |
| Presence of first aid facilities | 11 |
| Presence of emergency suitcase with required content | 17 |
| Presence of material for working hygienic | 9 |

| **Team** |  |
| --- | --- |
|  | Items |
| Executing technical and diagnostic tasks | 13 |
| Executing organizational en administrative tasks | 9 |
| Executing tasks with regard to chronic diseases and prevention | 11 |
| Having structured internal arrangements | 3 |
| Meetings within HAGRO (general practitioner’s group) | 2 |
| Having structured meetings with primary caregivers | 5 |
| Having structured meetings with health care organizations | 11 |
| Personnel policy | 1 |

| **Information** |  |
| --- | --- |
|  | Items |
| Medical reporting | 6 |
| Medical and non-medical information for patients | 8 |
| Presence of social map | 1 |

| **Quality and safety** |  |
| --- | --- |
|  | Items |
| Use of quality system with pharmacist | 1 |
| Working against standards and protocols | 2 |
| Quality policy | 3 |
| Education of practice employees | 5 |

**Patient experience**

| **Patient experience with general practitioner’s functioning from the previous 12 months*** | |
| --- | --- |
| **Items** | |
| 1 | Making you feel s/he had time during consultations |
| 2 | Interest in your personal situation |
| 3 | Making it easy for you to tell him/her about your problems |
| 4 | Involving you in decisions about medical care |
| 5 | Listening to you |
| 6 | Keeping your records and data confidential |
| 7 | Quick relief of your symptoms |
| 8 | Helping you to feel well so that you can perform your normal daily activities |
| 9 | Thoroughness |
| 10 | Physical examination |
| 11 | Offering you services for preventing diseases (e.g. screening, health checks, immunizations) |
| 12 | Explaining the purpose of tests and treatments |
| 13 | Telling you what you wanted to know about your symptoms and/or illness |
| 14 | Help in dealing with emotional problems related to your health status |
| 15 | Helping you understand the importance of following his or her advice |
| 16 | Knowing what s/he had done or told you during previous contacts |

*5-point Likert scale questions and an option ‘not applicable’

| **Patient experience with organization of care from the previous 12 months^*^** | |
| --- | --- |
| **Items** | |
| 1 | Preparing you for what to expect from specialists or hospital care |
| 2 | The helpfulness of the staff (other than the doctor) |
| 3 | Getting an appointment to suit you |
| 4 | Getting through to the practice by phone |
| 5 | Being able to speak to the GP by phone |
| 6 | Waiting time in the waiting room |
| 7 | Providing quick services for urgent health problems |
| **Accessibility in general practice and general practitioner^#^** | |
| 8 | It is possible to ask for a longer consultation |
| 9 | General practitioner is good accessible by phone |
| 10 | Patient gets another general practitioner regularly |
| **Procedure for complaints^#^** | |
| 11 | The practice has an accessible procedure for complaints |

*5-point Likert scale questions and an option ‘not applicable’

^#^Answering scale yes/no

**Un-incentivized clinical outcome indicators**

| **Diabetes** | |
| --- | --- |
| Information from the previous 12 months | |
| 1 | The percentage of patients in whom the Hba1c is 7 or less |
| 2 | The percentage of patients in whom the blood pressure is 150/85 or less |
| 3 | The percentage of patients whose measured total cholesterol is 5.0 mmol/l or less |

| **COPD** | |
| --- | --- |
| Information from the previous 12 months | |
| 1 | The percentage of patients with no exacerbation |

| **Asthma** | |
| --- | --- |
| Information from the previous 12 months | |
| 1 | The percentage of patients with no exacerbation |

| **Diabetes** | |
| --- | --- |
| Information from the previous 12 months | |
| 1 | The percentage of patients in whom the blood pressure is 160/90 or less |
| 2 | The percentage of high risk patients with statins whose measured cholesterol is 5.0 mmol/l or less |
